# Is Combination Antiviral Therapy Mandatory for Maintenance Therapy in Fully Suppressed Multidrug-Resistant Hepatitis B Patients?

**DOI:** 10.1155/2018/6948235

**Published:** 2018-12-16

**Authors:** Sung Won Chung, Young Chang, Hyo Young Lee, Eun Ju Cho, Jeong-Hoon Lee, Su Jong Yu, Jung-Hwan Yoon, Yoon Jun Kim

**Affiliations:** Department of Internal Medicine and Liver Research Institute, Seoul National University College of Medicine, Republic of Korea

## Abstract

**Aim:**

The efficacy of tenofovir disoproxil fumarate (TDF) monotherapy as maintenance therapy in multidrug-resistant (MDR) hepatitis B virus (HBV) patients after complete virologic suppression (CVS) has not been well evaluated. We evaluated the efficacy of maintenance TDF monotherapy compared with conventional TDF plus entecavir combination therapy after CVS of MDR HBV.

**Methods:**

In this single-center retrospective study, patients with MDR HBV who were previously treated with entecavir plus TDF combination therapy and achieved CVS were included. Patients were either maintained on entecavir plus TDF combination therapy or switched to TDF monotherapy after CVS. The primary endpoint was the virologic breakthrough, and secondary outcomes were liver cirrhosis (LC) or hepatocellular carcinoma (HCC) development. To overcome immortal time bias, time-varying Cox proportional hazard regression analysis was performed.

**Results:**

A total of 201 patients were included, and 153 patients were maintained on entecavir plus TDF combination therapy (combination group); 48 patients were converted from combination therapy to TDF monotherapy (single group) after CVS. Five patients experienced a virologic breakthrough, one patient in the single group owing to poor transient compliance and four patients in the combination group (*P* = 0.51). One new case of LC developed in the single group; five cases of LC developed in the combination group (*P* = 0.35). No new HCC development occurred in the single group, while seven cases of HCC developments were noted in the combination group. However, these results were not statistically significant (*P* = 0.54).

**Conclusions:**

For patients with suppressed HBV DNA, the efficacy of TDF monotherapy as maintenance therapy is comparable to that of entecavir plus TDF combination therapy.

## 1. Introduction

Treatment outcomes of patients with chronic hepatitis B (CHB) have improved since the development of nucleos(t)ide analogs (NAs), including lamivudine (LAM), adefovir (ADV), telbivudine (LdT), entecavir (ETV), or tenofovir disoproxil fumarate (TDF) [1–3]. Conversely, as the duration of NA treatment prolongs, the incidence of antiviral drug resistance increases, especially in patients using low potent and low genetic barrier NAs, including LAM or ADV. Many patients with single drug resistances are treated with sequential monotherapies. Consequently, multidrug-resistant (MDR) hepatitis B virus (HBV) has emerged [4, 5]. MDR HBV is of particular interest because MDR HBV has higher chances of increased viral loads, elevated serum alanine aminotransferase (ALT) levels, and development of liver cirrhosis (LC) or hepatocellular carcinoma (HCC) owing to viral control difficulties as MDR HBV has higher risks of resistance to other NAs [6–9].

Currently, most treatment guidelines recommend ETV plus TDF combination therapy for treating MDR CHB [10–13]. As TDF has a very high barrier to resistance, the possibility of TDF monotherapy successfully treating MDR CHB has increased. Recently, a randomized control trial comparing TDF monotherapy and ETV-TDF combination therapy in patients with LAM-resistant, ETV-resistant, and ADV-refractory CHB revealed no significant differences in HBV DNA < 15 IU/mL and virologic breakthroughs between the two treatment groups [10, 14, 15]. Based on this study, the Korean Association Study for the Liver and the American Association for the Study of Liver Diseases (AASLD) recommend TDF monotherapy as an alternative choice to treat MDR CHB [10, 12]. However, in a subgroup analysis, double ADV mutation (rtA181T/V and rtN236T mutation) showed significantly lower complete virologic suppression (CVS) rates [14, 15]. Therefore, for MDR HBV, especially in patients with ADV mutations, ETV-TDF combination therapy will be a safer option than TDF monotherapy [16]. Conversely, the cost-effectiveness of ETV-TDF combination therapy compared to TDF monotherapy needs further investigation [17]. Here, we clarified the safety of maintenance TDF monotherapy in patients with fully suppressed MDR CHB who were previously treated with ETV-TDF combination therapy.

## 2. Materials and Methods

### 2.1. Patient Selection and Inclusion and Exclusion Criteria

This single-center (Seoul National University Hospital, Seoul, Korea) retrospective study included consecutive patients diagnosed with MDR CHB, treated with ETV-TDF combination therapy for at least 6 months who reached a “no HBV DNA detected” status in the serum HBV viral load examination (<20 IU/mL). Patients with underlying malignancies besides HCC, previous history of liver transplantation, with antibodies against hepatitis C virus or human immunodeficiency virus, or previous exposure to TDF were excluded.

MDR was defined as “genotypic resistance” to two or more NAs or single ADV resistance mutation since ADV mutation is closely related to TDF mutation [4]. Genotypic resistance was defined as the identification of amino acid substitution mutations that confer LAM resistance (rtL180M/V or rtM204I/V/S), LdT (rtL180M + M204V, rtA181T/V, or rtM204I), ADV (rtA181T/V or rtN236T), or ETV (rtL180M + rtM204V/I ± rtI169T ± rtV173L ± rtT184A/G/L/S ± rtS202G/I ± M250 V) using direct polymerase chain reaction-based DNA sequencing methods [18]. Patients with previous history of LAM or LdT were evaluated separately.

Basic demographic information and laboratory findings were obtained at the index date. This study was approved by the institutional review board of the Seoul National University Hospital.

### 2.2. Follow-Up

The endpoint (virologic breakthrough, LC development, and HCC development) was recorded as follow-up duration. In almost every follow-up, HBV viral load, liver function test, and renal monitoring, which includes monitoring serum creatinine levels, estimated glomerular filtration rate (eGFR), and serum phosphate levels, were performed at 3-6-month intervals. Increases in HBV DNA by >1 log_10_ compared to nadir or in patients with previously undetected HBV DNA and HBV DNA elevations ≥100 IU/mL were defined as viral breakthroughs [12]. Minor virologic breakthrough was defined as any quantitative HBV DNA detection (HBV DNA ≥ 20 IU/mL). Imaging studies (liver sonography, dynamic abdomen computed tomography, or magnetic resonance imaging) were performed at least one time per year for HCC and LC surveillance.

LC diagnosis was based on the presence of at least one of the following criteria: (1) presence of stage F4 fibrosis in a liver biopsy; (2) detected portal hypertension, defined as a hepatic venous pressure gradient of least 6 mmHg, ascites detected in physical exam, or gastroesophageal varices detected in esophagogastroduodenoscopy; and (3) at least two signs of cirrhosis: nodular liver surface or portal vein diameter of >12 mm, spleen size of >12 cm, or platelet count of >100 k/mm^3^ in two consecutive imaging or laboratory studies [19–21]. HCC diagnosis was determined by tissue biopsy or typical imaging findings according to AASLD guidelines [22].

To evaluate renal complications, event of hypophosphatemia and chronic kidney disease (CKD) progression was checked in all patients. A hypophosphatemia event was defined as a serum phosphorous level lower than 2 mg/dL, and CKD progression was defined as new onset eGFR lower than 60 mL/min (CKD stage 3a) or stage progression of CKD stage 3a to 3b or higher. The CKD stage was defined in the Kidney Disease Improving Global Outcomes guideline [23].

### 2.3. Maintenance Monotherapy Conversion

When HBV DNA viral loads became undetectable (HBV DNA <20 IU/mL), conversion to monotherapy was performed. TDF was chosen as the monotherapy drug. The decision to incorporated TDF monotherapy depended on physicians' preference.

### 2.4. Data Analysis and Statistics

The index date was set at the date when CVS was achieved. Primary outcomes were viral breakthrough or minor virologic breakthrough after the index date. Secondary outcomes were the development of LC or HCC after the index date.

Categorical variable comparisons were performed using the chi-square or Fisher exact tests. Categorical variables were evaluated as proportions, and continuous variables were evaluated as medians (standard deviations). Continuous variable comparisons were performed using either the Mann-Whitney *U* test or the student *t*-test.

To compare viral breakthroughs and LC/HCC development, the Firth regression method was performed [24]. The initiation date of TDF monotherapy varied among patients. This different initiation date could lead to immortal time bias in clinical outcomes. To overcome immortal time bias, the time-varying Cox proportional hazards regression method was applied [25, 26]. Exposure of TDF monotherapy was treated as a time-dependent variable. Initially, TDF monotherapy patients were coded as the ETV-TDF combination group (the combination group) before the initiation of TDF monotherapy (defined as “pre-monotherapy time”) and after initiating TDF monotherapy, TDF monotherapy patients were coded as the TDF monotherapy group (the single group) [26]. If the number of clinical outcomes was less than ten events, multivariable analysis was not performed since multivariable analysis of data less than ten events per variable might lead to incorrect results [27]. All analyses were performed using R language version 3.43 (R Foundation for Statistical Computing, Vienna, Austria). *P* values of <0.05 were considered statistically significant.

## 3. Results

### 3.1. Baseline Characteristics

**Figure 1:**
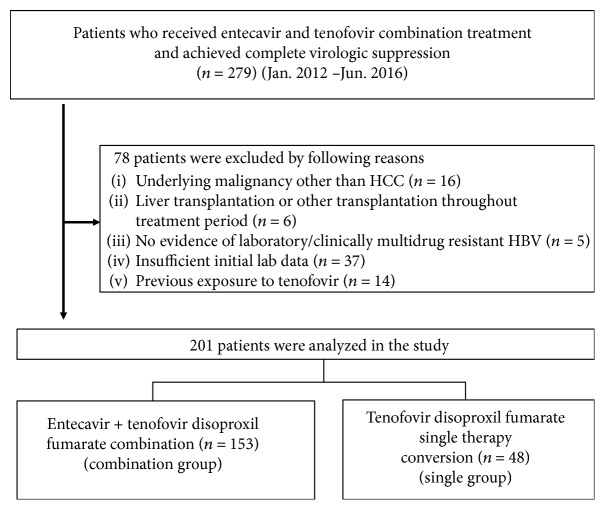
Flow diagram. A total of 279 patients were identified, and 78 patients were excluded by exclusion criteria. The final sample consisted of 201 patients, who were classified into 2 groups according to maintenance therapy (combination group versus single group).

Between January 2010 and June 2016, 201 patients received ETV-TDF combination therapy and met the eligibility criteria (Figure 1). Among the 201 patients, 153 were treated with maintenance ETV-TDF combination therapy (combination group) and 48 were treated with maintenance TDF monotherapy (single group). The median follow-up period was 47.7 months [interquartile range (IQR):34.6–53.4 months]. All patients had an experience in other NAs before ETV-TDF combination therapy, and 182 patients were exposed to LAM and 25 patients were exposed to LdT. The most common treatment just before ETV-TDF combination therapy was ETV-based therapy (*N* = 119, ETV single *n* = 60, ETV + ADV *n* = 59) followed by LAM and ADV combination therapy (*N* = 47). Baseline characteristics of the study population are represented in Table 1. There were no significant differences between the two groups except for the previous exposure to LdT (*P* = 0.003) (Table 1). The median time from CVS to TDF monotherapy conversion was 29.8 months (IQR 21.4–38.0 months), and the median treatment duration of the single group was 20.8 months (IQR 7.5–23 months). The median treatment duration of the combination group (including the pre-monotherapy time) was 42.5 months (IQR 28.9–52.0 months).

### 3.2. Comparison of Virologic Breakthroughs between Both Groups

During the study period, one case (2.1%) of virologic breakthroughs was observed in the single group, and four cases (3.2%) of virologic breakthrough were observed in the combination group. All events were transient (Supplementary Table S1), and most cases were related to a compliance issue (i.e., drug loss). There were no viral breakthrough events in the rtA181T/V and rtN236T double mutation patient group throughout the study period.

**Figure 2:**
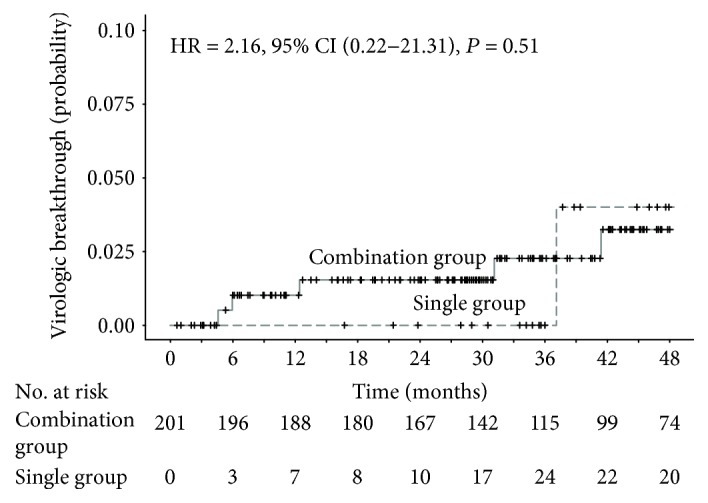
Extended Kaplan-Meier curves of cumulative incidence of virologic breakthrough, stratified by time-varying TDF monotherapy status. The index date was set as the date of complete virologic suppression. Hazard ratio (HR), 95% confidence intervals (CI), and *P* values were calculated by the time-varying Cox proportional hazard model with the time-varying covariate. CI: confidence interval; HR: hazard ratio; no.: number; TDF: tenofovir disoproxil fumarate.

Cumulative virologic breakthroughs were analyzed with the time-varying Cox proportional hazards regression model, and there were no significant differences between the two groups (hazard ratio [HR] = 2.16, 95%, confidence interval [CI] = 0.22–21.31, *P* = 0.51) (Figure 2, Table 2).

For further evaluation, a minor virologic breakthrough was evaluated; during the follow-up, 43 cases of minor virologic breakthrough were detected (3 cases in the single group and 40 cases in the combination group). Most cases occurred without definite cause, but all cases were transient. There were no significant differences between the two groups (HR = 1.12, 95% CI = 0.33–3.83, *P* = 0.85), and mutations did not affect minor virologic breakthroughs (Supplementary Table S2 and Figure S1). Positive HBeAg results were related to minor virologic breakthrough (HR = 2.92, 95% CI = 1.44–5.93, *P* = 0.002).

### 3.3. Subgroup Analysis of LC and HCC Development

**Figure 3:**
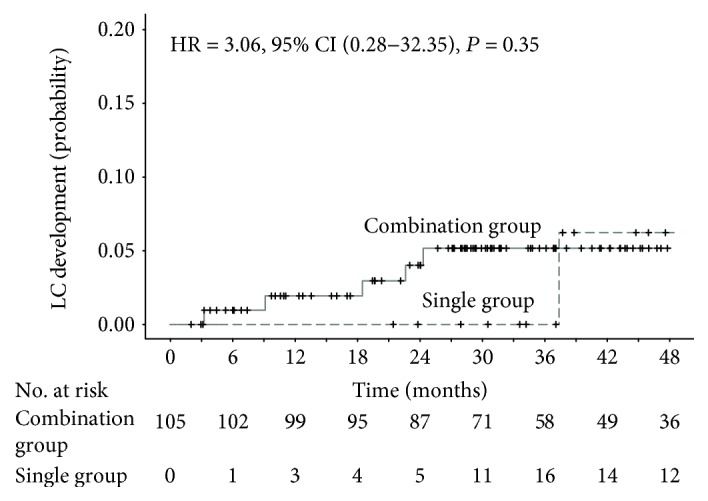
Extended Kaplan-Meier curves of cumulative incidence of liver cirrhosis development, stratified by time-varying TDF monotherapy status. The index date was set as the date of complete virologic suppression. Hazard ratio (HR), 95% confidence intervals (CI), and *P* values were calculated by the time-varying Cox proportional hazard model with the time-varying covariate. CI: confidence interval; HR: hazard ratio; LC: liver cirrhosis; no.: number; TDF: tenofovir disoproxil fumarate.

In total, 105 patients (combination group, *N* = 75; single group, *N* = 30) initially had noncirrhotic livers, and during treatment and follow-up, six patients (5.7%) were newly diagnosed with LC. One patient in the single group and five in the combination group were newly diagnosed with LC, but there were no significant differences between the two groups (HR = 3.06, 95%, CI = 0.28–32.35, *P* = 0.35) (Figure 3, Table 3).

**Figure 4:**
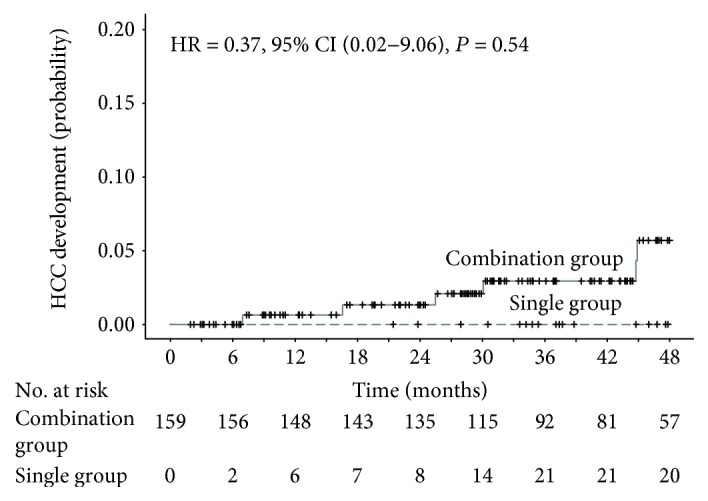
Extended Kaplan-Meier curves of cumulative incidence of hepatocellular carcinoma (HCC) development, stratified by time-varying TDF monotherapy status. The index date was set as the date of complete virologic suppression. Hazard ratio (HR), 95% confidence intervals (CI), and *P* values were calculated by the time-varying Cox proportional hazard model with time-varying covariate. CI: confidence interval; HCC: hepatocellular carcinoma; HR: hazard ratio; TDF: tenofovir disoproxil fumarate.

Initially, 159 patients had no previous history of HCC (combination group *N* = 117, single group *N* = 42), and during the treatment period, seven new cases of HCC (6.0%) were diagnosed. There were no new HCC developments in the single group and no significant differences between the two groups (HR = 0.37, 95% CI = 0.02–9.06, *P* = 0.54) (Figure 4). The baseline aspartate aminotransferase level was strongly related to HCC development (HR = 1.11, 95% CI = 1.07–1.16, *P* < 0.001) (Table 4).

### 3.4. Safety

Renal complication occurred in 16 patients (combination group, *N* = 14, single group, *N* = 2), and single therapy did not increase the risk of TDF monotherapy (HR = 1.95, 95% CI = 0.40–9.52, *P* = 0.41) (Supplementary Table S3). In multivariable analysis, high baseline creatinine significantly increase the risk of CKD progression (HR = 11.32, 95% CI = 1.37–93.27, *P* = 0.02).

Since ten patients had insufficient phosphorus data, 191 patients were analyzed for hypophosphatemia event (combination group, *N* = 144, single group, *N* = 47). Nineteen patients experienced hypophosphatemia (combination group, *N* = 16, single group, *N* = 3). TDF monotherapy did not increase the risk of hypophosphatemia (HR = 2.01, 95% CI = 0.53–7.64, *P* = 0.31) (Supplementary Table S4).

## 4. Discussion

This single-center retrospective study showed that in patients with MDR HBV who achieve CVS with combination antiviral therapies, the conversion to maintenance TDF monotherapy was comparable to continuous combination therapy in terms of HBV suppression and LC or HCC development without additional adverse events such as hypophosphatemia or CKD progression.

As the presence of MDR and high HBV DNA titer are known risk factors for CHB disease progression [2, 11], effective antivirals are especially important for treating the high viral load of MDR HBV. Lee et al. showed that after treating patients with MDR HBV with ETV-TDF combination therapy, 67.8–93.3% of patients achieved CVS at 12 months, irrespective of resistance profiles [16]. Recently, a randomized control trial showed that TDF monotherapy has comparable virologic responses at week 48 to ETV-TDF combination therapy [14]. Follow-ups to 144 weeks have been recently reported; no significant virologic breakthroughs in patients who received TDF monotherapy were reported, and 74.5% of patients achieved CVS in the TDF monotherapy group [15]. Thus, current treatment guidelines for MDR HBV can be divided into two methods. The first is to change combination therapies (i.e., TDF + ETV) [10–13], and the other is to switch to TDF monotherapies [10, 12]. Currently, there is no recommendation for how to treat patients with MDR HBV who achieve CVS.

Nam et al. recently reported that the rate of viral suppression was related to HCC or LC development [28]. Patients who failed to achieve CVS until 1 or 2 years showed higher incidences of HCC and LC compared with those who achieved CVS within 1 or 2 years [28]. As the viral load and time to CVS are related to LC and HCC development, for a rapid viral load reduction, combination therapy is more favorable than TDF monotherapy [14, 29].

In vitro, the rtA181T/V and rtN236T double mutation group showed a 10-fold reduction in susceptibility to TDF compared to patients infected with the wild-type virus [30]. Lim et al. reported that when patients with both rtA181T/V and rtN236T mutations were treated with TDF monotherapy, the viral HBV load decrease rate was significantly slower than that of patients treated with ETV-TDF combination therapy [14]. Chung et al. recently reported that patients who experienced ADV achieved less CVS and longer durations to CVS [29]. Both less-achieved CVS and prolonged durations to CVS favor ETV-TDF combination therapy, rather than TDF monotherapy.

Conversely, as TDF resistance is related to rtA181T/V and rtN236T mutations, TDF monotherapy has potential risks of TDF treatment failure in these patients. In our study, there were no virologic breakthrough events during the follow-up period in patients with rtA181T/V and rtN236T mutations, and neither rtA181T/V mutation nor rtN236 mutation was significantly associated with LC development or new HCC development. This result can be carefully interpreted and suggests that when HBV is completely suppressed with sufficient antivirals, TDF monotherapy can sufficiently prevent HBV viral breakthrough. Based on the previous and current studies, initially treating patients with MDR CHB with ETV-TDF combination therapy and changing the treatment regimen to TDF monotherapy after CVS may provide another treatment option, even in patients with reduced susceptibility to TDF mutations or patients previously treated with ADV.

The combination therapy did not increase drug-related adverse events [31], but increased economic burden is another issue among treated patients. Although generic drugs have been released, combination therapy doubles the cost. As life-long treatment of NAs is required, changing patients who are virally suppressed to TDF monotherapy will decrease the patient's economic burden by one half, without additional risks of LC or HCC development.

This study has a few limitations. First, this study was a retrospective cohort study; thus, patient selection and lack of clinical data with respect to bone loss complications derived from TDF were not completely evaluated in the study. Fortunately, previous studies that evaluated the risk of TDF complications found no significant increases in adverse events in TDF monotherapy and discovered that the continued use of single TDF did not increase complications compared to ETV-TDF combination therapies [15]. Tenofovir alafenamide is available worldwide, the use of which will further decrease TDF-related complications. Further investigations of the efficacy of tenofovir alafenamide as a single-agent maintenance therapy for MDR HBV is warranted.

Second, comparable long-term outcomes, such as LC or HCC development, between the single group and the combination group might have occurred by the small sample size of the single group or short follow-up period. Although there was no significant difference in the risk of virologic breakthrough, which is known to be related to LC or HCC development [32], between the single group and the combination group, long-term and large sample follow-up data might be needed for further validation.

## 5. Conclusions

Changing ETV-TDF combination therapy to TDF monotherapy after CVS is another treatment option for patients with MDR HBV, and there was no additional risk of viral breakthrough. There might have no significant difference in LC or HCC development compared to ETV-TDF combination therapy, but further long-term evaluation is needed for validation.

## Figures and Tables

**Table 1:** Demographic and baseline characteristics of the patients.

	Overall (*n* = 201)	Combination group (*n* = 153)	Single group (*n* = 48)	*P* value
Male (%)	153 (71.2)	111 (72.5)	33 (68.8)	0.74
Age, years (IQR)	53 (48–60)	54.0 (48–60)	51 (47–58.3)	0.44
HBeAg-positive (%)	117 (54.4)	79 (51.6)	29 (60.4)	0.37
Previous drug exposure, *n* (%)				
LAM	182 (84.7)	131 (85.6)	39 (81.3)	0.49
LdT	29 (13.5)	11 (7.2)	12 (25.0)	0.003
ETV	166 (77.2)	111 (72.5)	36 (75.0)	0.85
ADV	156 (72.6)	121 (79.1)	34 (70.8)	0.32
HBV-resistant mutations, *n* (%)				
To LAM + LdT	13 (6.5)	10 (6.5)	3 (6.3)	
To LAM + ETV	2 (1.0)	2 (1.3)	0 (0.0)	
To LAM + LdT + ETV	106 (52.7)	77 (50.3)	29 (60.4)	
To LAM + LdT + ADV	15 (7.5)	11 (7.2)	4 (8.3)	
To LAM + ETV + ADV	1 (0.5)	1 (0.7)	0 (0.0)	
To LAM + LdT + ETV + ADV	15 (7.5)	12 (7.8)	3 (6.3)	
To LdT + ADV	46 (22.9)	37 (24.2)	9 (18.8)	
To ADV only	3 (1.5)	3 (2.0)	0 (0.0)	
Mutation location, *n* (%)				
rtL180	141 (65.6)	96 (62.7)	35 (72.9)	0.23
rtA181	72 (33.5)	55 (35.9)	15 (31.3)	0.60
rtT184	65 (30.2)	42 (27.5)	20 (41.7)	0.07
rtS202	62 (28.8)	44 (28.8)	14 (29.2)	1.00
rtM204	158 (73.5)	107 (69.9)	39 (81.3)	0.14
rtN236	24 (11.2)	17 (11.1)	7 (14.6)	0.61
rtM250	6 (2.8)	3 (2.0)	3 (6.3)	0.15
rtA181 and rtN236	15 (7.0)	10 (6.5)	5 (10.4)	0.36
Underlying LC, *n* (%)	99 (46.0)	77 (50.3)	18 (37.5)	0.14
Previous history of HCC, *n* (%)	45 (20.9)	36 (2354)	6 (12.5)	0.11
Laboratory data, median (IQR)				
AST (IU/L)	27 (23–34)	27 (22–35)	27 (23–34)	0.37
ALT (IU/L)	26 (21–36)	26 (21–34)	26.5 (17.8–37)	0.21
Total bilirubin (mg/dL)	0.9 (0.7–1.2)	0.9 (0.7–1.2)	0.8 (0.6–1.2)	0.60
Albumin (g/dL)	4.4 (4.2–4.6)	4.4 (4.2–4.5)	4.4 (4.3–4.6)	0.46
Creatinine (mg/dL)	0.9 (0.8–1.0)	0.9 (0.8–1.0)	0.9 (0.8–1.0)	0.31
Phosphorous (mg/dL)	3.3 (3.0–3.6)	3.3 (2.9–3.6)	3.3 (3.1–3.5)	0.78

Data are *n* (%) or median (IQR). ADV: adefovir; ALT: alanine aminotransferase; AST: aspartate aminotransferase; ETV: entecavir; HBV: hepatitis B virus; HCC: hepatocellular carcinoma; IQR: interquartile range; LAM: lamivudine; LC: liver cirrhosis; LdT: telbivudine; SD: standard deviation.

**Table 2:** Univariable Cox proportional hazard analysis for virologic breakthrough (HBV DNA titer ≥ 100 IU/mL).

Parameter	Virologic breakthrough	
	HR (95% CI)	*P* value
Treatment		
Combination group	1 (reference)	
Single group	2.16 (0.22–21.31)	0.51
Age (per year)	0.97 (0.89–1.05)	0.45
Male	2.04 (0.24–17.29)	0.52
HBeAg		
Negative	1 (reference)	
Positive	3.93 (0.46–33.67)	0.21
Previous history of HCC		
Absent	1 (reference)	
Present	0.75 (0.09–6.43)	0.79
Underlying LC		
Absent	1 (reference)	
Present	1.11 (0.22–5.48)	0.90
AST (per 1 IU/L)	1.02 (0.97–1.07)	0.50
ALT (per 1 IU/L)	1.004 (0.96–1.05)	0.87
Total bilirubin (per 1 mg/dL)	0.38 (0.04–3.99)	0.42
Albumin (per 1 g/dL)	0.93 (0.11–8.20)	0.95
rtA181 mutation^∗^		
Absent	1 (reference)	
Present	0.38 (0.04–3.29)	0.38
rtN236 mutation^∗^		
Absent	1 (reference)	
Present	0.51 (0.004–4.34)	0.61
Minor virologic breakthrough		
Absent	1 (reference)	
Present	0.51 (0.02–12.22)	0.68

Time-varying Cox was applied for analysis. Virologic breakthrough was defined as HBV DNA titer ≥ 100 IU/mL. Minor virologic breakthrough was defined as HBV DNA titer ≥ 20 IU/mL. ^∗^Univariable factors were analyzed with Firth method. ALT: alanine aminotransferase; AST: aspartate aminotransferase; CI: confidence interval; HBV: hepatitis B virus; HCC: hepatocellular carcinoma; HR: hazard ratio; LC: liver cirrhosis.

**Table 3:** Univariable Cox proportional hazard analysis for LC development.

Parameter	Univariable analysis	
	HR (95% CI)	*P* value
Treatment		
Combination group	1 (reference)	
Single group	3.06 (0.28–32.35)	0.35
Age (per year)	1.04 (0.94–1.14)	0.44
Male	0.31 (0.06–1.54)	0.15
HBeAg		
Negative	1 (reference)	
Positive	2.92 (0.34–25.02)	0.32
AST (per 1 IU/L)	1.02 (0.93–1.12)	0.69
ALT (per 1 IU/L)	1.02 (0.98–1.06)	0.27
rtA181 mutation		
Absent	1 (reference)	
Present	0.35 (0.04–3.00)	0.34
rtA181 and rtN236 mutation		
Absent	1 (reference)	
Present	1.06 (0.05–23.64)	0.97
Virologic breakthrough		
Absent	1 (reference)	
Present	4.88 (0.12–207.22)	0.41
Minor virologic breakthrough		
Absent	1 (reference)	
Present	0.40 (0.02–9.64)	0.57

Time-varying Cox was applied for analysis. Virologic breakthrough was defined as HBV DNA titer ≥ 100 IU/mL. Minor virologic breakthrough was defined as HBV DNA titer ≥ 20 IU/mL. ALT: alanine aminotransferase; AST: aspartate aminotransferase; CI: confidence interval; HBV: hepatitis B virus; HR: hazard ratio; LC: liver cirrhosis.

**Table 4:** Univariable Cox proportional hazard analysis for HCC development.

Parameter	Univariable analysis	
	HR (95% CI)	*P* value
Treatment		
Combination group	1 (reference)	
Single group	0.28 (0.01–6.38)	0.42
Age (per 1 year)	1.06 (0.98–1.15)	0.13
Male	2.21 (0.27–18.38)	0.46
HBeAg		
Negative	1 (reference)	
Positive	0.47 (0.10–2.10)	0.32
Underlying LC		
Absent	1 (reference)	
Present	4.91 (0.95–25.30)	0.06
AST (per 1 IU/L)	1.11 (1.07–1.16)	<0.001
ALT (per 1 IU/L)	1.04 (1.02–1.07)	0.002
rtA181 and rtN236 mutation		
Absent	1 (reference)	
Present	3.58 (0.53–23.92)	0.19
Virologic breakthrough		
Absent	1 (reference)	
Present	1.60 (0.07–38.00)	0.77
Minor virologic breakthrough		
Absent	1 (reference)	
Present	1.08 (0.16–7.32)	0.93

Time-varying Cox was applied for analysis. Virologic breakthrough was defined as HBV DNA titer ≥ 100 IU/mL. Minor virologic breakthrough was defined as HBV DNA titer ≥ 20 IU/mL. ^∗^Univariable factors were analyzed with the Firth method. AST: aspartate aminotransferase; ALT: alanine aminotransferase; CI: confidence interval; HBV: hepatitis B virus; HR: hazard ratio; LC: liver cirrhosis.

## Data Availability

All relevant data are within the paper and its supplementary files.

## Abbreviations

AASLD: American Association for the Study of the Liver Diseases
ADV: Adefovir
ALT: Alanine aminotransferase
CHB: Chronic hepatitis B
CKD: Chronic kidney disease
CVS: Complete virologic suppression
ETV: Entecavir
HBV: Hepatitis B virus
HCC: Hepatocellular carcinoma
eGFR: Estimated glomerular filtration rate
LAM: Lamivudine
LC: Liver cirrhosis
LdT: Telbivudine
MDR: Multidrug-resistant
NA: Nucleos(t)ide analogs
TDF: Tenofovir disoproxil fumarate.

## Supplementary Materials

Supplementary Materials Figure S1: extended Kaplan-Meier curves of cumulative incidence of minor virologic breakthrough, stratified by time-varying TDF monotherapy status. Supplementary Table S1: patients' characteristics who experienced virologic breakthrough (≥100 IU/mL)/minor virologic breakthrough (≥20 IU/mL). Supplementary Table S2: univariable and multivariable Cox proportional hazard analysis for minor virologic breakthrough (HBV DNA titer ≥ 20 IU/mL). Supplementary Table S3: univariable and multivariable Cox proportional hazard analysis for chronic kidney disease progression. Supplementary Table S4: univariable and multivariable Cox proportional hazard analysis for hypophosphatemia.
