# Supplementary material for: Is Combination Antiviral Therapy Mandatory for Maintenance Therapy in Fully Suppressed Multidrug-Resistant Hepatitis B Patients?
Source: Gastroenterol Res Pract. 2018 Dec 16;2018:6948235. doi: 10.1155/2018/6948235 (PMC6311770; doi:10.1155/2018/6948235)
Supplement: Supplementary Materials — Figure S1: extended Kaplan-Meier curves of cumulative incidence of minor virologic breakthrough, stratified by time-varying TDF monotherapy status. Supplementary Table S1: patients' characteristics who experienced virologic breakthrough (≥100 IU/mL)/minor virologic breakthrough (≥20 IU/mL). Supplementary Table S2: univariable and multivariable Cox proportional hazard analysis for minor virologic breakthrough (HBV DNA titer ≥ 20 IU/mL). Supplementary Table S3: univariable and multivariable Cox proportional hazard analysis for chronic kidney disease progression. Supplementary Table S4: univariable and multivariable Cox proportional hazard analysis for hypophosphatemia. [file 6948235.f1.pdf]

Supplementary Materials

.

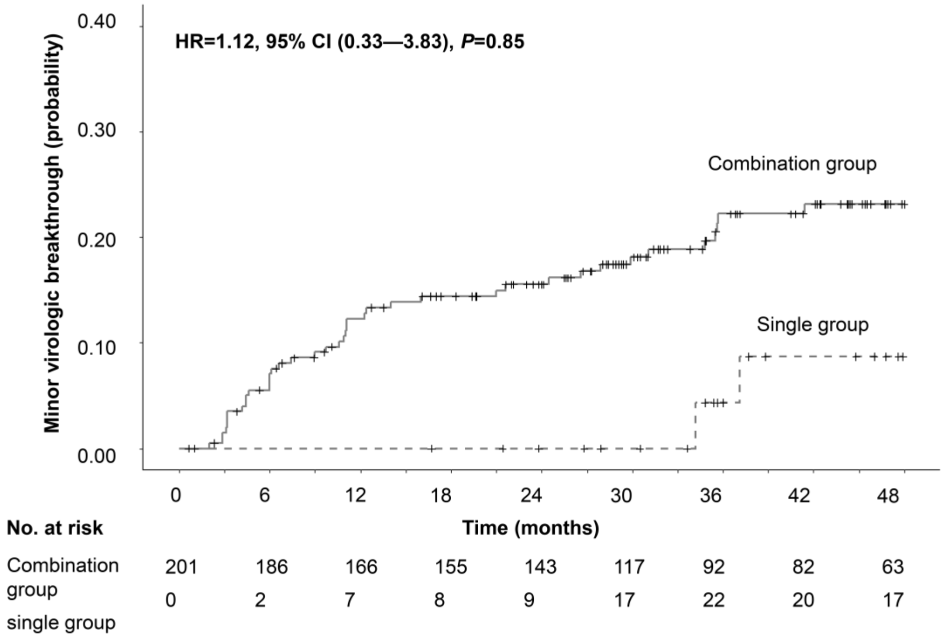

Supplementary Figure S1. Extended Kaplan-Meier curves of cumulative incidence of minor virologic breakthrough, stratified by time-varying TDF monotherapy status. The index date was set as the date of complete virologic suppression. Hazard ratio (HR), 95% confidence intervals (CI) and P values were calculated by the time-varying Cox proportional hazard model with time-varying covariate. Abbreviations: CI, confidence interval; HR, hazard ratio; TDF, tenofovir disoproxil fumarate.

Supplementary Table S1. Patients' characteristics who experienced virologic breakthrough (≥100 IU/mL) / minor virologic breakthrough (≥20 IU/mL)

No.	Group	Age	Sex	HBeAg	HBV DNA titer	ALT	Previous treatment before combination therapy	Months from combination treatment	Months from CVS	Clinical information	outcome	detected mutation
3	Com	47	F	+	650000	14	LAM+ADV	36.8	31.1	poor compliance	CVS	rtL180M, rtS202G, rtM204V
5	Com	55	F	_	20	30	ETV	14.1	10.6	poor compliance	CVS	rtL180M, rtM204V, rtM250V
7	Com	58	M	+	32	20	LAM+ADV	44.1	35.5		CVS	rtL180M, rtM204V, rtM250L
9	Com	43	M	+	170000000	17	ETV	54.2	41.4	poor compliance	CVS	rtL180M, rtS202G, rtM204V
10	Com	50	M	+	28	27	ETV+ADV	12.8	11.1		CVS	rtA181V

15	Com	61	M	+	1010	133	ETV	5.3	4.6		death‡	rtL180M, rtS202G, rtM204V
19	Com	55	F	+	53	24	ETV+ADV	9.9	4.4		CVS	rtA181T
26	Single	49	M	+	27	23	ETV+ADV	40.1	34.2		CVS	rtL180M, rtM204V, rtN236T
36	Com	54	M	-	28	17	LAM+ADV	17.6	4.4		CVS	rtA181T
39	Com	67	F	+	22	28	ETV+ADV	21.9	21.0		CVS	rtL80I, rtL180M, rtA181V, rtM204I, rtM204V, sG145R
41	Com	68	M	+	54	23	ETV+ADV	31.9	3.2		CVS	rtL180M, rtM204V
46	Com	40	M	+	64	81	ETV+ADV	38.0	6.1	poor compliance	CVS	rtL80I, rtM204I
47	Com	57	M	+	299	28	ETV	6.0	6.0		CVS	rtL180M, rtM204V, rtT184I, rtS202G

48	Com	61	F	+	31	29	ETV+ADV	7.9	3.2		CVS	rtL180M, rtM204I
49	Single	33	M	-	103	62	ADV	37.1	37.1	poor compliance	CVS	rtA181S, rtT184I, rtM204I
50	Com	53	M	-	36	40	LAM+ADV	25.4	9.0	poor compliance	CVS	rtL180M, rtS202G, rtM204V
55	Com	50	M	-	24	51	LAM+ADV	27.2	24.5		CVS	rtL180M, rtT184S, rtM204V
61	Com	49	M	+	37	44	LAM+ADV	29.9	29.9		CVS	rtL180M, rtM204I, rtM204V, rtS202G
70	Com	33	M	+	60	179	ETV+ADV	32.0	10.9	poor compliance	CVS	rtV173L, rtL180M, rtM204V, rtV173L
74	Com	48	M	+	21	48	LAM+ADV	18.4	12.3	poor compliance	CVS	rtA181T
79	Com	47	M	+	33	29	ETV	8.3	6.0		CVS	rtL180M, rtS202G, rtM204V

85	Com	63	M	+	30	31	ETV	22.1	14.0		CVS	rtA181T
86	Com	54	M	-	23	27	ETV+ADV	18.4	2.9		CVS	rtL180M, rtM204V
89	Com	57	F	+	46	37	ETV+ADV	8.5	2.9		CVS	rtL180M, rtT184A, rtS202G, rtM204V
91	Com	50	M	+	38	32	LAM+ADV	18.1	3.2		CVS	rtL80I, rtV173L, rtL180M, rtM204V
98	Com	63	M	+	1150	27	ETV	16.4	12.4		CVS	rtM204I
105	Com	50	M	+	63	36	LdT+ADV	7.9	4.2		CVS	rtA181T, rtM204I
106	Com	52	M	+	32	70	ETV+ADV	12.2	6.0		CVS	rtL80I, rtL180M, rtM204I
113	Single	63	F	+	20	22	LAM+ADV	59.0	57.4		CVS	rtL80V, rtA181T, rtM204I
138	Com	68	M	-	30	13	ETV+ADV	35.3	6.6		CVS	rtV173L, rtL180M, rtM204V

140	Com	44	M	-	21	27	ETV+ADV	22.4	3.1		CVS	rtL180M, rtA181T, rtM204V, rtN236T
158	Com	56	M	-	37	98	LAM+ADV	17.3	16.0		CVS	rtA181V
164	Com	63	F	+	21	152	ETV	28.9	26.6		CVS	rtA181S, rtT184I, rtM204I
165	Com	53	M	+	39	25	ETV	31.4	27.9		CVS	rtL180M, rtS202G, rtM204V
166	Com	62	F	+	50	84	LAM+ADV	38.4	35.7		CVS	rtV173L, rtL180M, rtM204V
174	Com	42	M	+	21	27	ETV+ADV	34.8	34.8		CVS	rtL80I, rtL180M, rtA181T, rtM204I
175	Com	29	M	+	32	48	ETV+ADV	14.5	7.4		CVS	rtA181T
180	Com	65	M	+	86	59	ETV	13.0	11.0		CVS	rtL180M, rtT184S, rtM204I

189	Com	51	M	+	29	20	ADV	16.8	11.1		CVS	rtA181V, rtN236T
192	Com	64	F	+	25	30	ETV	31.7	21.6		CVS	rtL80I, rtL180M, rtT184A, rtM204V
193	Com	71	M	+	22	30	ETV	39.1	35.6		CVS	rtL180M, rtT184A, rtM204V
203	Com	57	M	-	34	18	ETV	49.3	2.0		CVS	rtL180M, rtS202G, rtM204V
212	Com	52	F	+	41	20	ETV+ADV	15.3	9.7		CVS	rtA181V

Grey colored rows represent patients who experienced virologic breakthrough (HBV DNA titer ≥ 100 IU/mL) others represent minor virologic breakthrough (HBV DNA above the detection level which is HBV DNA titer ≥ 20 IU/mL). If there was clinical evidence of poor compliance in medical record, it was documented at the clinical information column. To detect HBV DNA mutation, PCR and direct sequencing method was applied. ‡ Patient expired due to hepatocellular carcinoma progression and before patient's death, CVS was achieved within the patient. Abbreviations: ADV, adefovir; CVS, complete virological suppression, ETV, entecavir; LAM, lamivudine; LdT, telbivudine.

Supplementary Table S2. Univariable and multivariable Cox proportional hazard analysis for minor virologic breakthrough (HBV DNA titer ≥ 20 IU/mL)

**Parameter**	**Univariable analysis**		**Mulivariable analysis**	
	HR (95% CI)	*P*-value	aHR (95% CI)	*P*-value
Treatment				
Combination group	1 [Reference]		1 [Reference]	
Single group	1.12 (0.33–3.83)	0.85	1.20 (0.35–4.15)	0.77
Age (per 1 year)	0.99 (0.97–1.03)	0.92		
Male	1.17 (0.59–2.32)	0.65		
HBeAg				
Negative	1 [Reference]		1 [Reference]	
Positive	2.92 (1.44–5.93)	0.002	2.93 (1.44–5.95)	0.003
Underlying LC				
Absent	1 [Reference]			
Present	0.77 (0.42–1.42)	0.41		

AST (per 1 IU/L)	1.01 (0.99–1.03)	0.28
ALT (per 1 IU/L)	1.01 (0.99–1.02)	0.25
Total bilirubin (per 1 mg/dL)	0.89 (0.52–1.53)	0.67
Albumin (per 1 g/dL)	0.85 (0.34–2.08)	0.72
Creatinine (per 1 mg/dL)	1.02 (0.23–4.55)	0.98
rtA181 and rtN236 mutation		
Absent	1 [Reference]	
Present	0.70 (0.23–2.54)	0.58

Time-varying cox was applied for analysis. *Univariable and multivariable factors were analyzed with Firth method. Abbreviation: aHR, adjusted hazard ratio; AST, aspartate aminotransferase; ALT, alanine aminotransferase; CI, confidence interval; HR, hazard ratio, LC, liver cirrhosis.

Supplementary Table S3. Univariable and multivariable Cox proportional hazard analysis for chronic kidney disease progression

Parameter	Univariable analysis		Mulivariable analysis	
	HR (95% CI)	*P*-value	aHR (95% CI)	*P*-value
Treatment				
Combination group	1 [Reference]		1 [Reference]	
Single group	1.95 (0.40–9.52)	0.41	1.65 (0.35–4.62)	0.99
Age (per 1 year)	1.05 (0.99–1.11)	0.05	1.04 (0.98–1.09)	0.17
Male	0.65 (0.24–1.80)	0.41		
Underlying LC				
Absent	1 [Reference]		1 [Reference]	
Present	2.53 (0.88–7.29)	0.08	2.02 (0.67–6.05)	0.21
Total bilirubin (per 1 mg/dL)	0.92 (0.42–1.99)	0.82		
Albumin (per 1 g/dL)	0.41 (0.18–0.91)	0.03		
Creatinine	4.21 (0.53–33.40)	0.17	11.19 (1.34–93.14)	0.03

(per 1 mg/dL)				
Phosphorous	2.29 (0.91–5.74)	0.08	3.12 (1.24–7.81)	0.02

Time-varying cox was applied for analysis. *Univariable and multivariable factors were analyzed with Firth method. Abbreviation: aHR, adjusted hazard ratio; CI, confidence interval; HR, hazard ratio, LC, liver cirrhosis.

Supplementary Table S4. Univariable and multivariable Cox proportional hazard analysis for hypophosphatemia

**Parameter**	**Univariable analysis**		**Mulivariable analysis**	
	HR (95% CI)	*P*-value	aHR (95% CI)	*P*-value
Treatment				
Combination group	1 [Reference]		1 [Reference]	
Single group	2.01 (0.53–7.64)	0.31	2.57 (0.58–11.39)	0.21
Age (per 1 year)	1.04 (0.99–1.09)	0.13		
Male	1.20 (0.43–3.34)	0.72		
Underlying LC				
Absent	1 [Reference]		1 [Reference]	
Present	5.48 (1.59–18.90)	0.007	5.37 (1.54–18.73)	0.008
Total bilirubin (per 1 mg/dL)	1.32 (0.95–1.85)	0.10		
Albumin (per 1 g/dL)	0.30 (0.17–0.54)	<0.001		
Creatinine	7.19 (0.95–54.08)	0.06	3.24 (0.51–20.65)	0.21

(per 1 mg/dL)				
Phosphorous	0.18 (0.07–0.48)	<0.001	0.23 (0.08–0.62)	0.004

Time-varying cox was applied for analysis. *Univariable and multivariable factors were analyzed with Firth method. Abbreviation: aHR, adjusted hazard ratio; CI, confidence interval; HR, hazard ratio, LC, liver cirrhosis.
